# Editorial: Insights in heart surgery: 2022

**DOI:** 10.3389/fcvm.2023.1184097

**Published:** 2023-04-17

**Authors:** Massimo Bonacchi, Beatrice Bacchi, Francesco Cabrucci, Hendrik Tevaearai Stahel, Robert Jeenchen Chen, Aleksander Dokollari

**Affiliations:** ^1^ Cardiac Surgery F.U., Experimental and Clinical Medicine Department, University of Florence, Firenze, Italy; ^2^ Clinic for Cardiovascular Surgery, Inselspital, University Hospital Bern, Bern, Switzerland; ^3^ Division of Cardiac Surgery, Department of Surgery, Ohio State University Wexner Medical Center, Columbus, OH, United States; ^4^ Department of Cardiac Surgery, Lankenau Institute for Medical Research, Main Line Health, Wynnewood, PA, United States

**Keywords:** advances and challenges, insight and practice, innovation, coronary revascularization surgery, valve repair and replacement, aortic surgery, HOCM

Editorial on the Research Topic Insights in heart surgery: 2022

Cardiac surgery continues to evolve over the years beyond current challenges, technologies, and “traditional” outcomes. This collection of articles “Insights into cardiac surgery” aims to highlight the latest advances in the field of cardiac surgery achieved during 2022.

Surgical approaches in cardiac surgery experienced a tremendous evolution in the last two decades. A lot of changes happened since the first operation performed by Goldwin et al. in 1958 (1) to treat hypertrophic obstructive cardiomyopathy (HOCM), including the advent of the Bentall technique for aortic root pathology, and the first coronary aortic bypass grafting (CABG), which today is the most commonly cardiac surgery procedure performed worldwide.

Different approaches have been previously described to treat HOCM (2). Raffa et al. introducing a method with the involvement of the subvalvular mitral apparatus [resection of anomalous muscular trabecula, accessory papillary muscles (PM), secondary chordae, and splitting of PM], showed excellent results including freedom from repeat intervention of 96% and significant symptomatic relief with NYHA and left ventricular obstruction reduction during midterm follow-up as well as a reduction in terms of mitral valve regurgitation incidence and septal thickness.

On the other hand, among aortic root repair strategies, Chang et al. suggested in case of pathological features of dissection, a sinus replacement technique using a patch trimmed to a scallop shape similar to Valsalva sinus, aiming to decrease severe aortic root bleeding. However, a cornerstone like the Bentall procedure created a race on describing the benefits of the modified technique in large clinical studies (3–5). In this context, Werner et al. closed the gap regarding long-term outcomes in patients undergoing the modified Bentall technique operation evidencing comparable results at 10-year follow-up with those of the general population. In this cohort of patients, the prosthesis choice in the so-called “gray area” (50–70 years old) is still controversial. Certainly, the advancement of transcatheter procedures (TAVR) with valve-in-valve aortic replacement and even the stentless valve prostheses, should be considered in the need of a reintervention (6) such as in the case of a “matryoshka procedure” (7). As a matter of fact, Chan et al. for aortic valve replacement (SAVR), underlined that the use of biological aortic prostheses has increased significantly in recent years in all age groups while mechanical valves are still higher in patients requiring dialysis. Although SAVR is an effective treatment with very low in-hospital mortality, in the last years, SAVR's rate is reducing especially in patients with high risk, octogenarians, and those requiring redo surgery due to the advent of TAVR.

Despite the spread-out of percutaneous coronary revascularization (PCI) CABG remains the most common cardiac surgery procedure worldwide and the best option for multivessel disease to achieve complete revascularization. Pasierski et al. highlight the importance of complete revascularization even in patients with pre-existing AF showing improved long-term survival and a lower rate of reinterventions. The advent of new technologies for CABG has been shown to be a benefit in improving the outcomes and increasing the heterogeneity of the patients. In this context, grafts’ availability is undoubtedly the first component needed to perform a CABG. In case of the lack of suitable autologous bypass material, Fusco et al. describe tissue-engineered vascular grafts (20 cm in length with an inner diameter of 3 mm) tested in animal models that showed good patency after 4 weeks.

Achieved the best available grafts, even their storage during the procedure, become crucial. Szalkiewicz et al. compare the use of saline with autologous blood vs. a preventive solution formulated with an endothelial damage inhibitor. The use of the second solution in the saphenous vein storage and testing during distal anastomosis has been described to be associated with lower levels of troponin after the procedure demonstrating superiority in preserving tissue functionality.

Beyond the surgical technique, in the current clinical practice, periprocedural risk predictors and optimization of medical therapy become fundamental before surgery to achieve a good outcome and to offer a tailored patient approach (8). For example, after tricuspid valve surgery (TVS) mortality remains high. In this particular group of patients, periprocedural risk predictors that impact long-term prognosis have not been fully investigated yet. Hasimbegovic et al. set the tone and paved the pathway to the adjustment of pre-procedural secondary prevention and optimization of medical therapy in patients undergoing TVS. Their “real world” study evidenced how patients with a high estimated plasma volume status (ePVS) have a significant impact on long-term outcomes after TVS. In this context, the author reported that the ePVS and Duarte's PVC were significantly lower in survivors. Risk predictors for long-term prognosis also included ePVS and gamma-glutamyltransferase levels.

Now more than ever, cardiovascular surgery feels the need to set a balance between adequate pre-operative patient medical optimization, the correct surgical procedure based on individual patient profiles, and the desire of treating complex conditions pushing forward the boundaries of the achievable. All of the articles in this Collection inspire, inform, and provide guidance and direction to researchers in the field, and could help us understand where cardiac surgery is going.

In conclusion, even if technology progresses by leaps and bounds significantly influencing surgical techniques and results, we must keep in mind that clinical success can be achieved only by multidisciplinary teamwork that adapts the chosen surgical strategy to the specific clinical profile of the individual patient.
